# Supplementary material for: Inhibition of Abl Kinase by Imatinib Can Rescue the Compromised Barrier Function of 22q11.2DS Patient-iPSC-Derived Blood–Brain Barriers
Source: Cells. 2023 Jan 27;12(3):422. doi: 10.3390/cells12030422 (PMC9913366; doi:10.3390/cells12030422)
Supplement: Supplementary file 1 [file cells-12-00422-s001.zip › Figure S1-3.pdf]

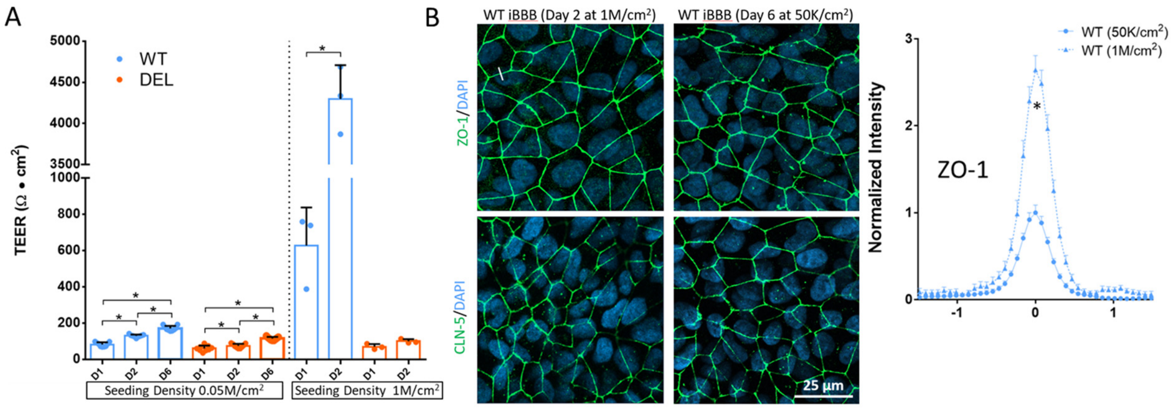

Figure S1 (A) Effects of seeding density of HBMECs on the TEER of the iBBB. * $p < 0.05$. Values are mean ± SD. (B) Effects of seeding density on the expression of tight junction proteins ZO-1 and claudin-5 of WT iBBB. * $p<0.05$.

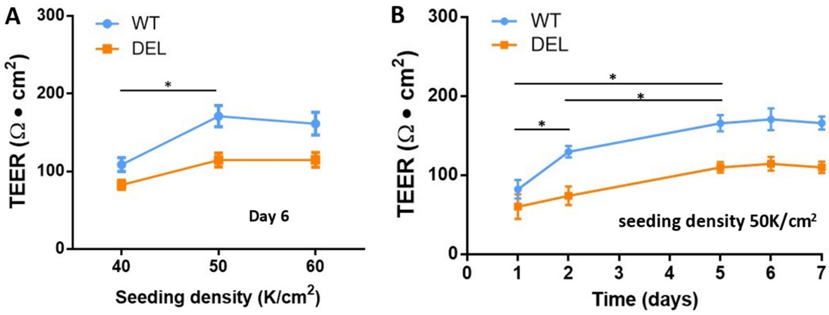

Figure S2 (A) Effects of seeding density of HBMECs on the TEER of the iBBB and (B) Effects of culturing time on the TEER of the iBBB. * $p<0.05$. Values are mean ± SD.

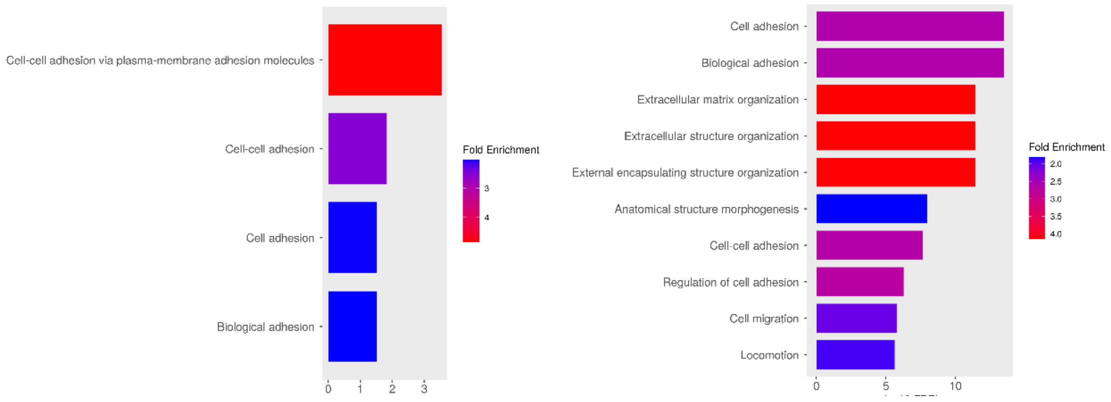

Figure S3 GO terms that are enriched in downregulated (left) and upregulated (right) DEGs between 22q11.2DS and controls without imatinib treatment
